# A new, high-resolution global mass coral bleaching database

**DOI:** 10.1371/journal.pone.0175490

**Published:** 2017-04-26

**Authors:** Simon D. Donner, Gregory J. M. Rickbeil, Scott F. Heron

**Affiliations:** 1 Department of Geography, University of British Columbia, Vancouver, British Columbia, Canada; 2 Integrated Remote Sensing Studio, Faculty of Forestry, University of British Columbia, Vancouver, British Columbia, Canada; 3 Coral Reef Watch, National Oceanic and Atmospheric Administration, Townsville, Queensland, Australia; 4 Global Science and Technology, Inc., Greenbelt, Maryland, United States of America; 5 Physics Department and Marine Geophysical Laboratory, College of Science, Technology and Engineering, James Cook University, Townsville, Queensland, Australia; King Abdullah University of Science and Technology, SAUDI ARABIA

## Abstract

Episodes of mass coral bleaching have been reported in recent decades and have raised concerns about the future of coral reefs on a warming planet. Despite the efforts to enhance and coordinate coral reef monitoring within and across countries, our knowledge of the geographic extent of mass coral bleaching over the past few decades is incomplete. Existing databases, like ReefBase, are limited by the voluntary nature of contributions, geographical biases in data collection, and the variations in the spatial scale of bleaching reports. In this study, we have developed the first-ever gridded, global-scale historical coral bleaching database. First, we conducted a targeted search for bleaching reports not included in ReefBase by personally contacting scientists and divers conducting monitoring in under-reported locations and by extracting data from the literature. This search increased the number of observed bleaching reports by 79%, from 4146 to 7429. Second, we employed spatial interpolation techniques to develop annual 0.04° × 0.04° latitude-longitude global maps of the probability that bleaching occurred for 1985 through 2010. Initial results indicate that the area of coral reefs with a more likely than not (>50%) or likely (>66%) probability of bleaching was eight times higher in the second half of the assessed time period, after the 1997/1998 El Niño. The results also indicate that annual maximum Degree Heating Weeks, a measure of thermal stress, for coral reefs with a high probability of bleaching increased over time. The database will help the scientific community more accurately assess the change in the frequency of mass coral bleaching events, validate methods of predicting mass coral bleaching, and test whether coral reefs are adjusting to rising ocean temperatures.

## Introduction

The fate of coral reefs on a warming planet has been the subject of great attention from scientists, governments, and the general public over the past few decades. Prolonged ocean temperatures of only 1–2°C above the range of usual coral experience can lead to the paling of reef-building animals due to a breakdown of the symbiosis with the colourful dinoflagellate *Symbiodinium* that reside in coral tissue [1]. Episodes of such mass coral “bleaching” around the world since the early 1980s have led to widespread coral mortality and raised questions about the viability of coral reef ecosystems during a period of rapid climate change [2] [3] [4]. Climate attribution research has found that anthropogenic forcing is likely (>90% chance) to drive recent mass bleaching episodes, including the extensive bleaching across the Eastern Caribbean in 2005 [5] and the northern Great Barrier Reef in 2016 [6]. Modeling studies suggest that projected ocean warming over the next three to four decades may make mass coral bleaching a frequent occurrence on most reefs worldwide, depending on assumptions about coral-symbiont acclimation and adaptation [7] [8] [9] [10].

Despite the overwhelming research attention and concern about mass coral bleaching, our knowledge of the extent of past bleaching episodes is greatly limited by geographical biases in the observational effort and the existing datasets cataloguing those efforts. The widely-used historical bleaching data available from ReefBase (reefbase.org) are limited by the voluntary nature of the submission to the database. Observations in ReefBase are clustered in more developed countries and areas of research interest, like the Caribbean and the Galapagos [11]. The available historical data therefore feature an unknown number of unobserved events or “false negatives.” In particular, the lack of data from the Pacific Ocean, home to the majority of the world’s coral reefs by area, severely limits the applicability of the data to: (i) global-scale analysis, (ii) enhancing real-time prediction methods, (iii) calibrating models for future prediction, or (iv) testing for acclimation or adaptation over time [11] [12]. Even in cases where bleaching reports are available, the uneven sampling effort creates geographic biases in the number and extent of recorded observations. For example, a 2002 bleaching event in Fiji, home to over 300 islands, has only two point reports in ReefBase, whereas an event that same year in Panama, which has only 10% the reef area of Fiji, has 65 reports. The number or spatial extent of available bleaching reports from ReefBase is therefore not a reliable measure of the change in the frequency of mass coral bleaching over time.

For this study, we developed a two-step process to address the shortcomings of the available global bleaching data. First, we developed a more comprehensive observational database of bleaching reports using targeted outreach to members of the international coral reef monitoring community and by searching the grey and academic literature. Second, we employed indicator kriging to develop annual high-resolution maps of the probability of bleaching occurrence from 1985 through 2010. We then analysed the new datasets to test for changes in the frequency of mass bleaching and the heat stress thresholds at which bleaching tends to occur. The products of this effort will be valuable for describing the extent of past bleaching events, testing bleaching prediction methods, and informing models that project the long-term response of coral reefs to ocean warming.

## Materials and methods

### Observational bleaching dataset

The new database follows the ReefBase format, including categories for source, country and site names, latitude and longitude of observation, year, month, percent bleached, percent mortality, depth, and survey method (Table 1). The percent bleached (and mortality, where available) is converted into a categorical variable following the same protocol as ReefBase (Table 2). While this simple method of bleaching reporting has many shortcomings, most notably no requirement for data on bleaching by taxa, it allows for consistency in reporting over time and for the inclusion of reports from rapid and low-technology bleaching assessments as well as those conducted by non-scientists.

**Table 1:** Bleaching database legend.

Category	Description
Country	Follows ReefBase convention
Location	State, region or island
Site_Name	Dive site or local community
Latitude	In decimal degrees
Longitude	In decimal degrees
Date	Date of observation *
Month	Month of observation *
Year	Year of observation
Depth	Depth of observation *
Severity_Code	See Table 2
Percent_Bleached	Percent of coral bleached
Mortality_Code	See Table 2
Percent_Mortality	Percent mortality, as a fraction of coral cover *
Survey_Type	Survey type (e.g., random swim, point intercept transect, etc) *
Source	Initial source of the report (i.e., dataset or group)
Name	Name of contributor of report to database (if relevant)
Citation	Source manuscript or report
Comments	Other comments on the record
Entry_Code	Researcher who entered the data
Database_Code	1 = ReefBase, 2 = New database

*if available

**Table 2:** Bleaching severity categories.

Level	Severity
-1	% unknown
0	No bleaching
1	Mild (1–10% bleached)^1^
2	Moderate (11–50% bleached)
3	Severe (>50% bleached)^1^

^1^ Mild and Severe are referred to as Low and High, respectively, in ReefBase

There were two stages in creating the database. First, the observational records in ReefBase were downloaded (for data through 2010, see “Thermal Stress and Data Analysis”) and subjected to a quality control procedure. If sufficient information was available, reports of non-warmwater bleaching (e.g., due to freshwater runoff or tidal exposure) and thermal bleaching caused by local events (warm power plant effluent) were removed [11]. In addition, reports with erroneous coordinates (bleaching reported on land) were corrected where possible using other location information and Google Earth, or otherwise removed.

Second, additional reports were collected from researchers and reef managers through a process of friendly coercion and literature research. This targeted “reef-by-reef” personal approach was used because of the typically low response rate to generic survey requests [13]. Research assistants and the lead author conducted a search of the Coral-List archives (available at http://coral.aoml.noaa.gov/pipermail/coral-list/) and the scholarly and grey literature (using Google Scholar, for publications from 1980 to 2012) for mentions of both “coral” and “bleaching.” Authors describing bleaching observations not or only partially recorded in ReefBase were then personally contacted for details on the event as well as any additional bleaching reports from other years or sites in their region of expertise. In addition, researchers and reef managers working in countries that were under-represented in ReefBase, the Coral-List archives, and the literature were personally contacted for missing observations.

For each data source, a personal email requesting data to help fill the specific geographical gap in ReefBase was sent to each contact, followed by a generic description of the database project. The recipients were offered to either share the raw information or to input the bleaching reports(s) directly via a template on our research group’s website. Each geographical coordinate with a unique bleaching observation was assigned a unique ID and thus included as an independent record in the database, provided that sufficient details were available (at minimum: the geographical coordinates, year of occurrence, and percent bleaching). Each new record in the database includes the name of the source and/or a literature citation.

The dataset ends in 2010 due to the time lag between bleaching occurrence and the availability of reports, as well as the availability of high-resolution historical sea surface temperature and thermal stress data reconstructed from satellite observations (see “Thermal Stress and Data Analysis”).

### Spatial modeling of bleaching occurrence

The probability of bleaching occurrence in a given year was spatially modelled across the world’s warmwater coral reefs at 0.04° × 0.04° latitude-longitude resolution using indicator kriging [14] [15]. This technique is designed to interpolate probabilities of occurrence of a binary condition, like the presence of a species, or in this case, a bleaching observation. Warmwater coral reef locations were extracted from the Millennium Coral Reef Mapping Project (UNEP-WCMC) [16]; all 0.04° × 0.04° grid cells containing reefs, regardless of attributes, were counted as reef cells in the model.

Bleaching presence in a given year was defined as any grid cell that contained at least one report of severity level 2 or 3 (>10% bleaching). Reports with lower or unknown bleaching severity were excluded because they are likely to represent non-lethal events or mistaken observations. Suggett and Smith [17] note misreading of non-lethal minor bleaching occurrences is a particular problem in voluntary monitoring and citizen science efforts, which were commonly the source of the original ReefBase data.

Pseudo-absences in a given year were defined as any reef cell for which the Degree Heating Week (DHW) values in 0.04° × 0.04° NOAA Coral Reef Watch data for the entire year were zero (see “Thermal Stress and Data Analysis”). The lack of positive DHW values indicates no thermal stress occurred that year. Thermal stress was otherwise not employed in the model. The interpolated bleaching probabilities therefore reflect only the observational data, the geography of the world’s coral reefs, and the lack of any thermal stress, not the magnitude of thermal stress or any other physical or biological variables. A subset (14%) of the observed bleaching reports had to be omitted from the indicator kriging procedure because of conflict between the coordinates of the observation, the cells on the coral reef maps, and/or the land mask in the Coral Reef Watch dataset.

For each year and region, empirical semi-variograms were assessed for range, sill, and nugget values after which eight different modeled semi-variograms (Exponential, Spherical, Gaussian, Matern, Stein's Matern, Circular, Linear, Bessel, Pentaspherical) were fit [18]. The model with the lowest Root Mean Squared Error was selected and used to estimate the bleaching probability in each grid cell. In cells with a raw bleaching observation but annual maximum DHW of 0°C-weeks, the estimated bleaching probability will be less than 1; the kriging procedure records both a bleach point and a pseudo-absence point, and the estimated probability for that grid cell, as for all other cells, must be calculated following the selected model. No indicator kriging was carried out if there were no reports that year or all eight of the modeled semi-variograms failed to converge due to the low number of bleaching observations that year. In those cases, the interpolated bleaching probability is zero for all cells, regardless of whether bleaching was observed.

The kriging procedure was carried out separately for the Caribbean, Indian Ocean, the main Pacific Ocean, and the Eastern Pacific Ocean in each year. The Eastern Pacific (e.g., Galapagos and Central America) was treated separately because of the distance from other Pacific coral reefs. To reduce computational requirements, the main Pacific Ocean was split into three sections with 1000 kilometres of overlap between each section; estimated bleaching probabilities were averaged in these overlapping sections to eliminate arbitrary edge effects from splitting the domain.

The resulting 0.04° × 0.04° gridded probability maps are presented here. The maps were also re-projected onto the initial reef polygon map from the Millennium Coral Reef Mapping Project to calculate the area of reefs with different probabilities of bleaching in each year. Regional analysis was conducted using regions defined by Kleypas et al. [19].

### Thermal stress data and analysis

To determine which reef locations were exposed to thermal stress and in which years, we used satellite-derived temperature data, following Heron et al. [20] and briefly described here. The Pathfinder version-5.2 dataset [21] is a NOAA Climate Data Record for sea surface temperature. From this, thermal stress for the period 1985–2010 was determined by the DHW metric [22], which combines the magnitude and duration of anomalous warm temperatures. Reef locations were identified as stressed in each year that the DHW value was greater than 0°C-weeks. The annual maximum value is used because the bleaching reports do not consistently contain sufficient information about the timing of bleaching onset and peak in the observational database; for example, 15% of the data in ReefBase do not report the month of observation at all.

## Results

### Observational database

**Fig 1:**
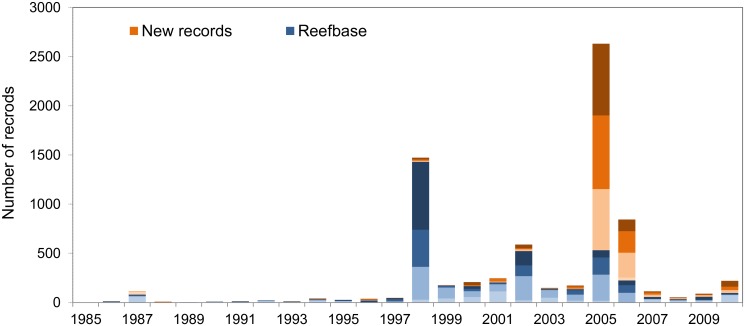
Annual number of bleaching observations for 1985–2010. Blue bars represent original ReefBase reports; orange bars represent new reports. Shading reflects bleaching level, from mild (1–10%) or unknown extent of bleaching (levels 1 and -1 in Table 2, lightest colour) to severe (>50%) bleaching (level 3, darkest colour).

After quality control procedure and removal of no bleaching reports, there were 4146 independent bleaching reports in the ReefBase data through 2010 (Table 3). The new observational dataset has 7429 reports, an increase of 79% from the ReefBase total. Two-thirds (67%) of the new reports are bleaching level 2 and 3, in contrast to 50% of the ReefBase reports of this severity. The highest number of new bleaching reports (64%) come from the Caribbean in 2005 where there was extensive bleaching [23] that was not well documented in ReefBase. Due to the additional reports, 2005 emerges as the year with the highest number bleaching observations, rather than 1997/1998 from ReefBase alone (Fig 1). There are also substantial increases in the number of reports from Indo-Pacific countries under-represented in ReefBase. For example, reports from Kenya (22 to 59 reports) and Fiji (56 to 114) are more than doubled in the new database, while reports from Kiribati reports increase from only 2 in ReefBase to 39 in the new database.

**Table 3:** Summary of observational bleaching data.

Observations		Total	Severe	Moderate	Mild	Unknown
ReefBase	All years	4146	1169 (28%)	907 (22%)	1406 (34%)	664 (16%)
	1998	1431 (35%)	690	378	335	28
	2005	533 (13%)	75	175	266	17
New Records	All years	3283	1062 (32%)	1137 (35%)	1001 (30%)	83 (3%)
	1998	42 (1%)	19	12	10	1
	2005	2098 (64%)	729	748	618	3
Total	All years	7429	2231 (30%)	2044 (28%)	2407 (32%)	747 (10%)
	1998	1473 (20%)	709	390	345	29
	2005	2631 (35%)	804	923	884	20

The new database shows a dramatic increase in the number of bleaching observations over time. Bleaching reports begin in the 1960s, but no single year has more than 20 observations until 1982, or more than 100 observations until 1987. There are only 12 observations of bleaching at any level before 1980; this increased to 236 during the 1980s, 1874 during the 1990s, and 5094 during the 2000s. If only observations of moderate to severe (level 2 and 3) bleaching are considered, there are just two reports before 1980, 63 during the 1980s, 1232 during the 1990s, and 2863 during the 2000s.

**Fig 2:**
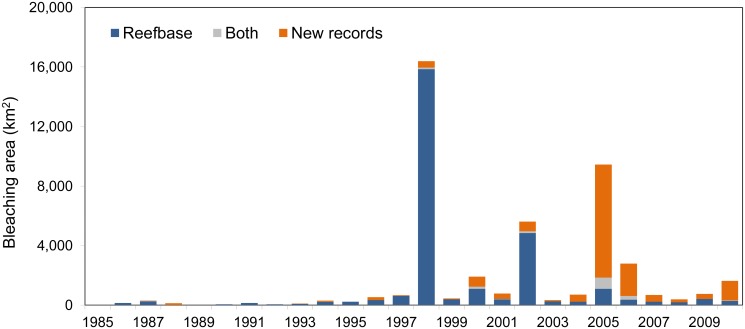
Annual area of moderate and severe (level 2 and 3) bleaching observations for 1985–2010. The area is computed from 0.04° × 0.04° latitude-longitude grid cells containing original ReefBase reports (navy blue), new records (orange), and those in both datasets (grey).

**Fig 3:**
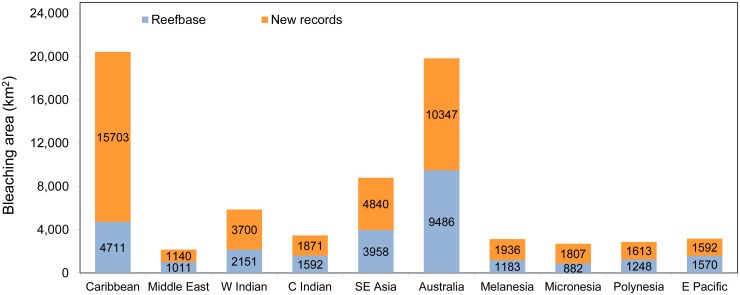
Total area (km^2^) of moderate and severe (level 2 and 3) bleaching by region over the 1985–2010 period. The area is computed based on 0.04° × 0.04° latitude-longitude grid cells representing original ReefBase reports (blue) and new reports (orange).

Due to uneven sampling effort and the geography of coral reefs, the number of observations is a potentially misleading measure of the extent of bleaching in a given year. Gridding the data onto the 0.04° × 0.04° resolution indicates that the spatial extent of bleaching observations was highest in 1998, rather than 2005 (Fig 2). Though numerous, the new 2005 reports are clustered in the eastern Caribbean with multiple reports per grid cell. The gridded data suggest that new reports represent a 61% increase in the reported area of cells with bleaching (over ReefBase), with 50% of that increase occurring in 2005, 14% in 2006, and 9% in 2010. Integrating over the entire time period (Fig 3), the largest relative increase in observed bleaching occurred in the Caribbean (333% increase), followed by Micronesia (205%), Western Indian Ocean (172%), Melanesia (164%), Polynesia (129%), and Southeast Asia (122%). The area of observed bleaching grid cells in Australia, though only doubling the previous number, increased by over 10 000 km^2^.

### Interpolated bleaching probabilities

The number of bleaching observations was sufficient to compute interpolated maps of bleaching probabilities for 18 years within 1985 through 2010 (Table 4). In 1985, 1986, and 1989–1994 there were either no bleaching reports (i.e., 1985, 1989) or too few bleaching reports for the modeled semi-variograms to converge in all regions (Fig 1). In eight of 18 years, interpolations could only be performed in one region; only two years (1998 and 2002) had sufficient observations to interpolate within five or six of the regions (Table 4).

**Table 4:** Years for which indicator kriging was conducted.

Year	Caribbean	Indian Ocean	East Pacific	Main Pacific Ocean			Any Regions
				East	Central	West	
1985	-	-	-	-	-	-	-
1986	-	-	-	-	-	-	-
1987	X	-	-	-	-	-	X
1988	X	-	-	-	-	-	X
1989	-	-	-	-	-	-	-
1990	-	-	-	-	-	-	-
1991	-	-	-	-	-	-	-
1992	-	-	-	-	-	-	-
1993	-	-	-	-	-	-	-
1994	-	-	-	-	-	-	-
1995	X	-	-	-	-	-	X
1996	-	X	-	-	-	-	X
1997	-	-	X	-	-	-	X
1998	X	X	X	X	X	X	X
1999	X	-	-	-	-	-	X
2000	-	-	-	X	X	X	X
2001	-	-	-	X	-	X	X
2002	X	X	-	X	X	X	X
2003	X	-	-	-	-	-	X
2004	X	X	-	-	X	-	X
2005	X	X	-	-	-	-	X
2006	X	-	-	-	-	-	X
2007	X	X	-	X	-	-	X
2008	X	-	-	X	-	-	X
2009	X	-	-	X	-	X	X
2010	-	X	-	-	-	X	X

**Fig 4:**
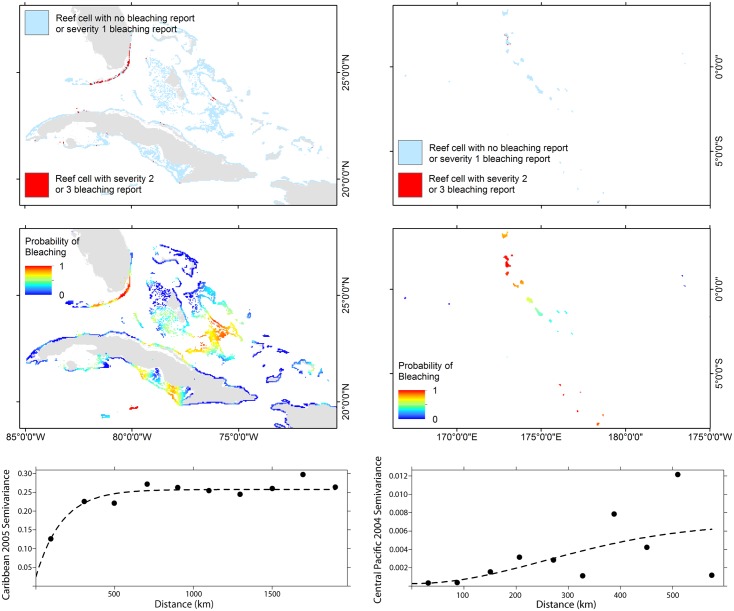
Examples of bleaching observations and probabilities for the Caribbean in 2005 (a,c,e) and central-west equatorial Pacific in 2004 (b,d,f). Top panels (a,b) show the raw observations of moderate and severe bleaching (level 2 and 3) from the database; middle panels (c,d) show the interpolated bleaching probabilities; and bottom panels (e,f) show the semi-variograms for the region and year.

In each of these 18 years, indicator kriging expanded the historical representation of the area that likely experienced bleaching. This is demonstrated in maps (Fig 4) for 2005 in northeastern Caribbean, where coral reef monitoring is common and reporting was relatively dense [23], and for 2004 in the central equatorial Pacific around Kiribati and Tuvalu, where coral reef monitoring is rare and reporting was opportunistic [24]. The spatial relationship between the raw observations in these two examples is characterized by their semi-variograms (Fig 4e and 4f), which describe the dissimilarity between values as a function of distance. The best fit model (dashed line) was used to estimate the bleaching probability in each cell in that region and year. In regions and years for which the interpolation did not succeed due to low density of observations, bleaching probabilities were set to zero across the region, which likely results in an underrepresentation of the bleaching probability.

**Fig 5:**
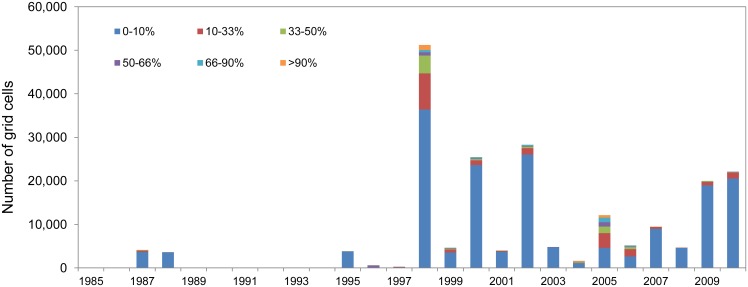
Number of 0.04° × 0.04° latitude-longitude grid cells with different estimated probabilities of bleaching from 1985–2010. Note that the density of bleaching reports was too low in years 1985, 1986, and 1989–94 for the interpolation to succeed.

Out of the 18 years in the 1985–2010 period for which indicator kriging could be conducted, 18, 17, and 13 years had cells with bleaching being more likely than not (>50% probability), likely (>66% probability), and very likely (>90% probability), respectively (Fig 5). Projecting the gridded bleaching probabilities onto the global coral reef map shows that the mean fraction of global coral reef area with a more likely than not chance of bleaching in any given year from 1985–2010 is 0.5%. This fractional area of probable bleaching varies from a low of zero in years with no reports or too few reports to perform indicator kriging, to highs of 3.3% in 2005 and 4.2% in 1998.

Regionally, the interpolated probabilities indicate the average relative extent of bleaching from 1985–2010 was greatest in the Eastern Pacific (3.3% of coral reef area per year with a more likely than not probability), the Central Indian Ocean (2.4%), and Caribbean (2.0%), and lowest in Melanesia (0.2%) and the Middle East (0.2%). The most extensive bleaching by region occurred in the Central Indian Ocean in 1998 (66% of reef area with a more likely than not chance of bleaching), followed by the Eastern Pacific Ocean in 1998 (52%), the Caribbean in 2005 (28%), and Micronesia in 2004 (15%).

Using the available data, there is no trend over time in the number of grid cells or the coral reef area with >50%, >66%, and >90% bleaching probability, or in the mean bleaching probability of all reefs. However, if the 1985–2010 probability database is divided into the 12-year periods before (1985–1996) and after (1999–2010) the 1997/1998 El Niño and worldwide bleaching event, there is a 8-fold, 8-fold, and 3-fold increase in the average area of coral reefs with bleaching probability >50%, >66%, and >90%, respectively, between the earlier and later periods. The increase in the area of reef is significant (p = 0.05, t-stat = -2.17, df = 12 for a two-tailed t-test assuming unequal variances) for probability >50%, but not significant for probability >66% (p = 0.07, t-stat = -1.95, df = 12) or >90% (p = 0.3, tstat = -1.07, df = 15) for which there are fewer years to sample.

### Bleaching and thermal stress

To test for a relationship between thermal stress and mass coral bleaching, we compared the DHW value for all reef cells with the DHW of cells with a high probability of bleaching. Over the 1985–2010 period, the reef area-weighted mean and median of the annual maximum DHW of reef cells with more likely than not, likely, or very likely probabilities of bleaching were each significantly higher (two-sided t-test, p<0.01) than the mean and median DHW of all reef cells (Table 5). The mean (and median) annual maximum DHW of reef cells that likely and very likely bleached (8.01°C-weeks and 8.10°C-weeks respectively) is similar to the NOAA Coral Reef Watch threshold for Bleaching Alert Level II (8°C-weeks), at which severe bleaching and some mortality is expected. It is important to note, however, that the annual maximum DHW described here refers to the highest value reached during that calendar year, which may be higher than the value at which bleaching began or became severe.

**Table 5:** Thermal stress for reefs with different bleaching probabilities.

Annual maximum DHW (°C-weeks)	All reefs	>90%	>66%	>66–90%	>50–66%
Area-weighted	1.71	8.16^	8.01*^	7.87*^+^	6.69*^+^
Median	<0.01	8.26^	8.10*^	7.92*^+^	6.49*^+^

* significantly different (<0.05 level) from probability of >90%

^+^ significantly different (<0.05 level) from probability of >66%

^ significantly different (<0.05 level) from probability of >66–90%

There were also significant differences in the area-weighted mean of annual maximum DHW for coral reefs with different probabilities of bleaching (Table 5). The annual maximum DHW of reefs (area-weighted average 8.16°C-weeks) with a very likely (>90%) probability of bleaching was significantly greater than that of reefs with a likely to very likely (>66–90%) probability of bleaching (7.87°C-weeks; p<0.001, t-stat = 16.05, df = 3298) and of reefs with a more likely than not to likely (>50–66%) probability of bleaching (6.69°C-weeks; p<0.001, t-stat = 17.66, df = 3733). There was also a significant difference between annual maximum DHW of reefs with a >50–66% bleaching probability with that of all reefs with a >66% bleaching probability (p<0.001, t-stat = 8.22, df = 4539), but not with that of reefs with a >66–90% bleaching probability (p = 0.51, t-stat = 0.66, df = 5561).

The temporal variation in thermal stress and interpolated bleaching probabilities provide a window into the possible changes in susceptibility of coral reefs to thermal stress over time.

**Fig 6:**
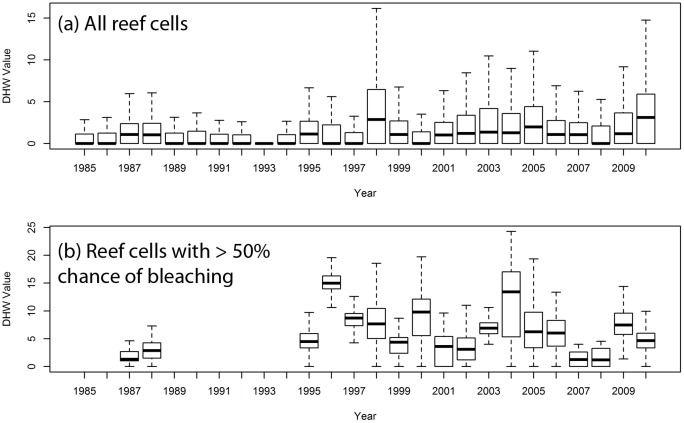
Mean, quartile range, and 5^th^ and 95^th^ percentile of reef area-weighted annual maximum DHW of a) all reef cells and b) reef cells with at least 50% probability of bleaching (more likely than not) from 1985–2010. Note that the density of bleaching reports was too low in years 1985, 1986, and 1989–94 for the interpolation to succeed.

A test of whether the threshold at which bleaching occurs has increased over time (due to adaptation, acclimation, or loss of susceptible taxa) is if the DHW of reefs that experienced bleaching increased faster than that of all reefs, or the surface ocean in general. Annual maximum DHW was averaged across all reef cells for each year, weighted by the coral reef area present in each cell. This area-weighted mean of annual maximum DHW for all reef cells increased significantly (p<0.01) over the 26-year period, by 0.08°C-weeks per year (2.14°C-weeks over the entire period; Fig 6a). The total increase was greatest in the Caribbean (4.60°C-weeks), Middle East (3.98°C-weeks), and Melanesia (2.18°C-weeks). There was, however, no significant increase over time in the area-weighted mean of annual maximum DHW of reef cells with more likely than not (Fig 6b), likely, or very likely probability of bleaching.

There were slight differences in the rate of change for DHW of reefs that experienced bleaching with that of all reefs between the 12-year period (1985–1996) before the 1997/1998 El Niño event and that after the event (1999–2000). The mean of annual maximum DHW for all reefs rose by 118%, from 1.02°C-weeks in 1985–1996 to 2.23°C-weeks in 1999–2010 between the two periods. The mean annual maximum DHW for reefs with a likely (>66%) probability of bleaching rose by slightly less, by 103%, from 3.82°C-weeks to 7.77°C-weeks, whereas that of reefs with a very likely (>90%) probability of bleaching increased by slightly more, by 146%, from 3.69°C-weeks to 9.07°C-weeks. The differences between the two periods were all highly significant (p<0.001).

Analysis of the trend in DHW at the regional level is limited by the lower number of years for which individual regions have cells reporting non-zero probability of bleaching. In the Caribbean, for which there are 11 years of data, the mean annual maximum DHW of reefs with a very likely probability (>90%) increased by 152%, from 3.08°C-weeks to 7.75°C-weeks, between the 12-year periods before and after the 1997/1998 El Niño. The increase in DHW between the two time periods was 132% and 119% for likely (>66%) and more likely than not (>50%) probability of bleaching respectively. By contrast, the area-weighted DHW of all Caribbean reefs increased nearly four-fold (from 0.89°C-weeks to 3.37°C-weeks) between the two time periods.

## Discussion

The expanded global observational bleaching database provides insight into the patterns in mass coral bleaching over time and the relationship between bleaching and thermal stress. It can also be applied to examine the influence of other factors like thermal history [25] [20] and reef resilience [26]. While the ReefBase voluntary bleaching database has been a valuable service for coral reef researchers and managers for many years, the data mining effort undertaken for this study suggests that, without ongoing curation, a voluntary database can suffer from substantial data gaps. The new observational database developed here includes 79% more bleaching records. Notably, two-thirds of the new records are moderate (11–50% coral bleached) or severe (>50% coral bleached) bleaching observations, which more reliably reflect large-scale thermal events than low-intensity observations [17].

Clustering of bleaching reports in areas of high monitoring effort remains an issue in the expanded database. Rasterizing the data partly controls for the uneven sampling effort and provides a consistent measure (area or number of grid cells) of the extent of mass coral bleaching. The difference between the number of reports in a given year (Fig 1) and the number of 0.04° × 0.04° cells with reports in a given year (Fig 2) demonstrates that a straightforward count of the number of bleaching reports is a potentially misleading metric (e.g., contrasting 1998 and 2005).

Expanding and rasterizing the observed bleaching database allowed for spatial interpolation or extrapolation to coral reefs where no monitoring was conducted. The calculated fraction of coral reef area with a more likely than not chance of bleaching (p>50%), estimated by projecting the gridded probability data onto the UNEP-WCMC coral reef map, is generally lower than that of other estimates of the extent of past regional- or global-scale bleaching events. For example, this analysis suggests 4.2% of coral reefs worldwide had a more likely than not chance of bleaching in 1998, whereas the oft-quoted and misquoted 2000 Status of the Coral Reefs of the World Report summary of the 1997–1998 event states that “approximately” 16% of the world’s coral reefs not only suffered bleaching but died during the event [27]. Such discrepancies may result for various reasons. Methodological differences exist between the indicator kriging performed here and the less spatially explicit geographical extrapolation employed in past studies. Here, the area of bleaching will be zero in any region and year in which the interpolation did not succeed due to low density of observations. In addition, this study used the newer UNEP-WCMC coral reef map that includes large areas of reef that feature few corals, like the largely sand lagoons of most Pacific atolls; this inflates the global reef area and adds locations that are unlikely to have observed bleaching records to inform the spatial modelling. For these reasons, this study’s estimate for 1985–2010 of 0.5% annual frequency of bleaching being more likely than not (p>50%)–implying that, in any given year during that period, 0.5% of the world’s coral reefs had a more likely than not chance of bleaching—could be low.

The lack of a significant increasing trend in the extent of bleaching in the interpolated dataset reflects the large year-to-year variability in thermal stress, bleaching extent, and observational effort (relative to the length of the time series), rather than the lack of a change in bleaching extent over past decades. First, an enormous increase in bleaching observations began in the early 1980s; only 12 of the 7436 bleaching reports (0.16%) in the new observational database are from before 1980. The increase is highly unlikely to be from sampling effort alone. Second, given that the canonical or “super” El Niño events which cause surface ocean temperature anomalies and mass coral bleaching in multiple ocean basins only occur roughly once every 20 years [28], a single event like 1997/1998 can obscure a long-term trend in the extent of coral bleaching. If the interpolated bleaching probabilities are examined at decadal time scales, an increase in the extent of bleaching is more readily apparent. The area of reef with a more likely than not (>50%) or likely (>66%) probability of bleaching was eight times higher after than before the 1997/1998 El Niño.

Extension of the observations database and the interpolated bleaching probabilities through the 2014–2016 “global” bleaching event [29,30] may further indicate a decadal-scale increasing trend in the extent of mass coral bleaching. However, it should be noted that although ocean temperatures and bleaching-level thermal stress are projected to continue rising even in an aggressive mitigation scenario [7, 9, 10], it is possible that bleaching observations will not increase in the near future due to the decline of susceptible taxa or populations. Bleaching occurrence is influenced by the composition of the coral and symbiont community. Reefs that experience more recent bleaching may be less likely to experience subsequent moderate to severe bleaching, despite the occurrence of repeat thermal stress, due to loss of coral cover, thermal acclimatization and/or shifts in community composition [31, 32, 33].

The results do show a close historical relationship between the occurrence of thermal stress and a high probability of bleaching, similar to that used by the Coral Reef Watch program in near real-time bleaching prediction. The annual maximum DHW was significantly higher (difference of 1.47°C-weeks) for coral reef cells with a very likely (>90%) probability of bleaching than those with a more likely than not to likely (>50–66%) probability. Most notable, the annual maximum DHW for coral reef cells with a likely or very likely chance of bleaching was approximately equivalent to the Bleaching Alert II threshold (8°C-weeks) used by the Coral Reef Watch program to predict severe bleaching with possible mortality. This correspondence should be viewed with caution because this study employed the maximum DHW value from the calendar year, rather than the DHW at the time of bleaching occurrence because the latter is usually not available in the dataset (e.g., except in the cases of dedicated monitoring programs, observations of bleaching may be weeks or months after the initial occurrence, or not report the specific date or month). Since the annual maximum DHW will often be higher than the value at which bleaching occurs or become severe, the ~8°C-weeks value reported here is likely an overestimate of the mean DHW at the onset of bleaching from 1985–2010.

The results also indicate that annual maximum DHW of coral reefs with a high probability of bleaching increased over time. Since the oceans are warming and thermal stress is increasing, an increase in the annual maximum DHW of coral reefs experiencing bleaching may indicate that temperature extremes have become more intense, but is not in itself evidence of adaptation or acclimation to rising temperatures. However, there is some evidence in the data that the annual maximum DHW of coral reefs with a high probability of bleaching (>90%) increased more rapidly over time than the annual maximum DHW of all coral reefs; if correct, that difference may suggest some adjustment to rising temperatures, whether by acclimation of corals or loss of susceptible species. Notably, the results suggest the reverse in the Caribbean, with annual maximum DHW increasing less rapidly over time in coral reefs with a high probability of bleaching, suggesting no or more limited adjustment to rising temperatures. There has been a marked decline in reef-building corals in the Caribbean [34] and resilience to disturbances, including thermal stress, in the Caribbean is thought to be low [35].

While the interpolated bleaching probabilities provide a means to test for trends in the extent of bleaching over time and how the relationship between thermal stress and bleaching may adjust over time, the accuracy of the interpolated data is still limited by the coverage of the observational database and the quality of the original reports. First, in years and regions with a lack of moderate and severe bleaching reports, whether due to the lack of actual bleaching or a lack of observations, the spatial interpolation was not possible. Second, many of the early reports from ReefBase feature limited information, including unknown causes of bleaching, and unknown extent of bleaching, which potentially introduce errors by being included (or omitted). In addition, some reports had to be excluded from the analysis due to unresolvable conflicts between the coordinates of the reported observation and the land mask for the 0.04° × 0.04° grid. These problems were mitigated somewhat by focusing on the more reliable moderate and severe reports; the trade-off is that fewer records limited the information available for the kriging analysis. Finally, in order to control for differences in sampling and reporting methods, the observational bleaching reports are currently limited to the simple measure of percent coral bleaching (and mortality) used by ReefBase; taxa-level data would help more specifically test the drivers of changes in bleaching occurrences and thresholds over time [36].

## Conclusion

The new databases of coral bleaching observations and the probability that bleaching occurred for the 1985–2010 period can support research on the effects of climate change on coral reefs. The databases are available at http://www.simondonner.com/bleachingdatabase as well as via the public data repository figshare (“Coral Bleaching Database V1”). The analysis of the new data demonstrates the increase in coral bleaching over the past three decades, the relationship between coral bleaching and thermal stress at a global scale, and the potential utility of such global databases for validating bleaching prediction methods and testing for changes in bleaching resilience at large-scales. Further work will be necessary to expand the coverage of the observed database, including adding new bleaching observations since 2010 and further backfilling the database with older missing reports, and thus better inform the spatial modeling of bleaching probabilities.
